# Supplementary material for: Large order fluctuations, switching, and control in complex networks
Source: Sci Rep. 2017 Sep 6;7:10663. doi: 10.1038/s41598-017-08828-8 (PMC5587719; doi:10.1038/s41598-017-08828-8)
Supplement: Supplementary file 1 — Supplementary Information [file 41598_2017_8828_MOESM1_ESM.pdf]

Supplementary Information (SI): Large order fluctuations, switching, and control in complex networks

Jason Hindes[1,*] and Ira B. Schwartz[1]
[1]U.S. Naval Research Laboratory, Code 6792, Plasma Physics Division, Nonlinear Systems Dynamics Section, Washington, DC 20375
[*]jason.hindes.ctr@nrl.navy.mil

1 Master equation expansion

We want to find the leading contribution to Eq.(3)(main text) when $C \gg 1$, where C is the number of stochastic realizations of the dynamical process defined by Eq.(1)(main text). Taylor expanding the probability and rates we find

$$P(\mathbf{m} \pm \frac{2}{C}\mathbf{1}_i, t) = ae^{-CS(\mathbf{m}\pm\frac{2}{C}\mathbf{1}_i,t)} \approx ae^{-C\left[S(\mathbf{m},t)\pm\frac{2}{C}\frac{\partial S}{\partial m_i}(\mathbf{m},t)+\ldots\right]} = ae^{-CS(\mathbf{m},t)}e^{\mp 2p_i}, \tag{1}$$

$$R_i^+(\mathbf{m} - \frac{2}{C}\mathbf{1}_i) \approx R_i^+(\mathbf{m}) + \left[\frac{1}{1+e^{-2\beta\sum_j A_{ij}m_j}} + f_i\right], \tag{2}$$

and

$$R_i^-(\mathbf{m} + \frac{2}{C}\mathbf{1}_i) \approx R_i^-(\mathbf{m}) + \left[\frac{1}{1+e^{2\beta\sum_j A_{ij}m_j}} + f_i\right]. \tag{3}$$

Substituting these approximations into Eq.(3)(main text) gives:

$$\begin{aligned} &-C\frac{\partial S}{\partial t}ae^{-CS(\mathbf{m},t)} = Cae^{-CS(\mathbf{m},t)}H(\mathbf{x},\mathbf{p}) \\ &\sum_i ae^{-CS(\mathbf{m},t)}e^{2p_i}\left[\frac{1}{1+e^{-2\beta\sum_j A_{ij}m_j}} + f_i\right] + ae^{-CS(\mathbf{m},t)}e^{-2p_i}\left[\frac{1}{1+e^{2\beta\sum_j A_{ij}m_j}} + f_i\right] \end{aligned} \tag{4}$$

where $H(\mathbf{m},\mathbf{p})$ is given by Eq.(6)(main text). Dividing by C and $ae^{-CS(\mathbf{m},t)}$, and neglecting the $\mathcal{O}(1/C)$ sum, we find Eq.(5)(main text).

2 Equilibria and linear spectra

In general Eqs.(8-9)(main text) have three equilibria, $\mathbf{m}=\mathbf{0}$ and $\pm\mathbf{m}^*$, that satisfy $\dot{\mathbf{m}}=\dot{\mathbf{p}}=\mathbf{0}$ with $\mathbf{p}=\mathbf{0}$. In the special case where the spontaneous flipping rate is homogeneous, $f_i = f\ \forall i$, we find a set of fixed-point conditions:

$$m_i^* = \frac{\tanh\left(\beta\sum_j A_{ij}m_j^*\right)}{1+2f}. \tag{5}$$

Often, it is convenient to approximate A by the largest term in its eigenvalue decomposition, i.e., a large spectral-gap assumption. When A is symmetric, $A = A^T \approx \lambda\boldsymbol{\eta}\boldsymbol{\eta}^T$, where η_i is the eigenvector centrality of node i and λ is the largest eigenvalue. Hence, a single equation determines the ordered equilibria (states) in terms of the order-parameter, $\overline{m}^* = \sum_i \eta_i m_i^* / \sum_j \eta_j$:

$$\overline{m}^* = \frac{\sum_i \eta_i \tanh\left(\beta\lambda\overline{m}^*\eta_i\sum_j\eta_j\right)}{[1+2f]\sum_l \eta_l}, \tag{6}$$

where $\sum_i \eta_i^2 = 1$. The linear stability spectra of the equilibria are found by substituting $\mathbf{m} = \mathbf{m}^* + \boldsymbol{\epsilon}$ and $\mathbf{p} = \boldsymbol{\mu}$ into Eqs.(8-9)(main text) and solving to $\mathcal{O}(\epsilon)$ and $\mathcal{O}(\mu)$. Because the resulting equations are linear, the dynamics are exponential: $\epsilon(t) = \epsilon e^{\sigma t}$ and $\mu(t) = \mu e^{\sigma t}$. The linearized dynamics gives three equations for the exponent σ and the relative size (shape) of the modes ϵ_i and μ_i:

$$\sigma(\mathbf{m}^*) = 1 + 2f - \beta\lambda\sum_j \eta_j^2\left[1 - (1+2f)^2 m_j^{*2}\right], \tag{7}$$

$$\mu_i = M\eta_i, \tag{8}$$

and

$$\epsilon_i = \frac{\mu_i(1+2f)}{(\sigma+1+2f)}\Big[2(1-m_i^{*2}) + \frac{\beta\lambda}{\sigma}\big[1-(1+2f)^2 m_i^{*2}\big]\sum_j \eta_j^2[1-m_j^{*2}]\Big], \tag{9}$$

where M is an arbitrary constant.

Ordered states emerge at a threshold where the equilibrium ($\mathbf{m}=\mathbf{0}$, $\mathbf{p}=\mathbf{0}$) changes stability, $\sigma(\mathbf{0})=0$:

$$\beta\lambda = 1+2f. \tag{10}$$

When $\beta\lambda > 1+2f$, ordered sates have $\sigma(\mathbf{m}^*)>0$ and $\sigma(\mathbf{0})<0$ for the modes Eqs.(8-9). However, the mean-field assumptions implicit in our approach can be quantitatively inaccurate for the threshold depending on the network. However, the WKB approach can be augmented to include pairwise correlations, for example, which generally improves accuracy. We mention that other solutions are possible with $\mu_i \equiv 0$, which have oppositely signed spectra in terms of stability, i.e., where $\mathbf{m}=0$ is unstable and $\mathbf{m}=\mathbf{m}^*$ is stable. In general, taking $\mathbf{p}\equiv\mathbf{0}$ $\forall t$ in Eqs.(8-9)(main text) gives the so called "quenched mean field" equations corresponding to a dynamic Ising model with random flipping. In this way, the condition $\mathbf{p}\neq\mathbf{0}$ is what allows a trajectory (i.e, the OP) to exist from a "stable" to "unstable" state in the deterministic mean-field theory.

3 Network details

The Facebook network used throughout the paper was taken from http://snap.stanford.edu/data/egonets-Facebook.html. It contains 4039 nodes and 88234 edges. The power-law network in Fig.2(a)(main text) was generated from the configuration model with degree(k) distribution, $g_k = k^{-2.5}/\sum_{k'=10}^{300} k'^{-2.5}$, and 600 nodes. In Fig.4(a)(main text) the Erdős-Rényi network had 500 nodes and 15000 edges and the bimodal network was generated from the configuration model with 400 nodes and two degree classes: 40 nodes had degree 50 and 360 nodes had degree 5.

4 Near threshold OP

As in the main text, we consider the special case where $f=0$ near threshold, with $\delta = \beta\lambda - 1 \gtrsim 0$. Our approach is to find $\mathbf{m}^*$ and the linear dynamics (Eqs.(7-9)) near $\mathbf{m}\approx\mathbf{0}$ and $\mathbf{m}\approx\mathbf{m}^*$ to lowest order in δ. This will give us boundary conditions which can be used to determine the OP to the same order in δ. Once the OP is found, we can explicitly perform the line integral of momentum that gives the Action, Eq.(7)(main text), and hence the probability exponent in the distribution of large fluctuations, Eq.(4)(main text). For this section, it is *not assumed that A has a large spectral gap nor is symmetric.*

First, we expand Eq.(5) in powers of δ, $m_i = \delta^{1/2} m_{i,1} + \delta^{3/2} m_{i,2} + ...$, and collect terms of the same order in δ:

$$\mathcal{O}(\delta^{1/2}): \qquad m_{i,1} = \frac{1}{\lambda}\sum_j A_{ij} m_{j,1}, \tag{11}$$

$$\mathcal{O}(\delta^{3/2}): \qquad m_{i,2} = m_{i,1} - \frac{1}{3} m_{i,1}^3 + \frac{1}{\lambda}\sum_j A_{ij} m_{j,2}. \tag{12}$$

Eq.(11) implies $m_{i,1} = E\eta_i$, and taking the inner product of Eq.(12) with the left eigenvector, ζ_i, corresponding to η_i, gives $E = \sqrt{3/\sum_j \zeta_j \eta_j^3}$, or

$$m_i^* = \eta_i \delta^{1/2}\sqrt{3/\sum_j \zeta_j \eta_j^3} + \mathcal{O}(\delta^{3/2}). \tag{13}$$

Note: $\sum_i \zeta_i \eta_i = 1$.

Next, we find the dynamics near $\mathbf{m}\approx\mathbf{0}$. Analogous to Eqs.(7-9) (without the symmetric A assumption),

$$-\sigma(\mathbf{0})\mu_i(\mathbf{0}) = -\mu_i(\mathbf{0}) + \frac{1+\delta}{\lambda}\sum_j A_{ji}\mu_j(\mathbf{0}), \tag{14}$$

$$\sigma(\mathbf{0})\epsilon_i(\mathbf{0}) = -\epsilon_i(\mathbf{0}) + 2\mu_i(\mathbf{0}) + \frac{1+\delta}{\lambda}\sum_j A_{ij}\epsilon_j(\mathbf{0}). \tag{15}$$

Solving Eqs.(14-15) we find that $\epsilon_i = \mathcal{E}\eta_i$ and $\mu_i = -\delta\mathcal{E}\zeta_i$, which means that

$$\frac{\mu_i(\mathbf{0})}{\epsilon_i(\mathbf{0})} = \frac{dp_i}{dm_i}(\mathbf{0}) = -\frac{\delta\zeta_i}{\eta_i}, \tag{16}$$

or the derivative at the boundary $\mathbf{m}=\mathbf{0}$ and $\mathbf{p}=\mathbf{0}$. Similarly, we seek the derivative at the boundary $\mathbf{m}=\mathbf{m}^*$ and $\mathbf{p}=\mathbf{0}$ to the same order, $\mathcal{O}(\delta)$. The linearized dynamics are

$$-\sigma(\mathbf{m}^*)\mu_i(\mathbf{m}^*) = -\mu_i(\mathbf{m}^*) + \frac{1+\delta}{\lambda}\sum_j A_{ji}\mu_j(\mathbf{m}^*) - \frac{3\delta}{\lambda\sum_l \zeta_l\eta_l^3}\sum_j A_{ji}\mu_j(\mathbf{m}^*)\eta_j^2 + \mathcal{O}(\delta^2), \tag{17}$$

$$\begin{aligned}\sigma(\mathbf{m}^*)\epsilon_i(\mathbf{m}^*) = -\epsilon_i(\mathbf{m}^*) + \left[2 - \frac{3\delta\eta_i^2}{\sum_l \zeta_l\eta_l^3}\right]\mu_i(\mathbf{m}^*) + \frac{1+\delta}{\lambda}\sum_j A_{ji}\epsilon_j(\mathbf{m}^*) - \frac{3\delta\eta_i^2}{\lambda\sum_l\zeta_l\eta_l^3}\sum_j A_{ji}\epsilon_j(\mathbf{m}^*)\\ + \mathcal{O}(\delta^2),\end{aligned} \tag{18}$$

Solving Eqs.(17-18) gives $\epsilon_i = \mathcal{A}\eta_i$ and $\mu_i = 2\delta\mathcal{A}\zeta_i$, implying a derivative boundary condition

$$\frac{\mu_i(\mathbf{m}^*)}{\epsilon_i(\mathbf{m}^*)} = \frac{dp_i}{dm_i}(\mathbf{m}^*) = \frac{2\delta\zeta_i}{\eta_i}. \tag{19}$$

Finally, it is convenient to parameterize m_i and p_i in terms of a unit-length parameter h, such that $h \equiv m_i/m_i^* \;\forall i$. Note: when $h=1$ the network is ordered at $\mathbf{m}^*$, and when $h=0$ the network has no order $\mathbf{m}=\mathbf{0}$. Therefore we can write, $m_i(h) = m_i^* h$ and $p_i(h) = \delta m_i^*\zeta_i f(h)/\eta_i$, where $f(h)$ is an unknown function that we must determine. The boundary conditions above imply: $f(h=1)=0$, $f(h=0)=0$, $\frac{df}{dh}(h=1)=2$, and $\frac{df}{dh}(h=0)=-1$. If we assume that $f(h)$ is a polynomial, the simplest polynomial that satisfies the four boundary conditions is a cubic function, $f(h) = h\big(h-1\big)\big(h+1\big)$. Hence we arrive at Eqs.(10-12)(main text). We mention that the near threshold OP is a convenient initial guess for the Iterative-Action-Minimization-Method described in Sec.6.

5 Scaling away from threshold

We would like to use Eqs.(7-9) to find basic scalings of the OP away from threshold, where the network's metastable order is high, which will help us understand fluctuations near $\mathbf{m}\approx\mathbf{0}$ and $\mathbf{m}\approx\mathbf{m}^*$ – i.e., the largest and smallest fluctuations. We first study the former for which the solution of Eqs.(7-9) is useful: $\mu_i/\epsilon_i = [1+2f-\beta\lambda]/[1+2f] = \frac{dp_i}{dm_i}(\mathbf{0})$. Therefore, the momentum is linear in m_i with constant slope across the network, $p_i \approx m_i[1+2f-\beta\lambda]/[1+2f]$. By considering the action at $\mathbf{m}=\mathbf{0}$, $S(\mathbf{0}) = \sum_i\int_{m_i}^0 p_i(m_i^{'})dm_i^{'} + \sum_i\int_{m_i^*}^{m_i} p_i(m_i^{'})dm_i^{'} = \sum_i\int_{m_i}^0 p_i(m_i^{'})dm_i^{'} + S(\mathbf{m})$, or $-S(\mathbf{m}) = -S(\mathbf{0}) - \sum_i\int_0^{m_i} p_i(m_i^{'})dm_i^{'}$, we find

$$-S(\mathbf{m}) \approx -S(\mathbf{0}) + \left[\frac{\beta\lambda - 1 - 2f}{1+2f}\right]\sum_i\int_0^{m_i} m_i^{'}dm_i^{'} = -S(\mathbf{0}) + \left[\frac{\beta\lambda-1-2f}{1+2f}\right]\sum_i\frac{m_i^2}{2}. \tag{20}$$

Using Eq.(19) we derive the relative probabilities for the very largest fluctuations, i.e., the tail of the large-fluctuation distribution to small $\mathbf{m}$, or Eq.(13)(main text).

A similar technique gives the small fluctuations near global consensus, $\mathbf{m}\approx\mathbf{m}^* \lesssim \mathbf{1}$. Assuming $f\approx 0$ and $A\approx\lambda\boldsymbol{\eta}\boldsymbol{\eta}^T$, Eq.(9) gives $\epsilon_i/\epsilon_j = [1-m_i^{*2}]/[1-m_j^{*2}]$. In this region, $m_i^{*2}\approx 1-4e^{-2\beta\lambda\eta_i\sum_j\eta_j m_j^*}$, and therefore

$[m_i - m_i^*]/[m_j - m_j^*] \sim [\eta_i/\eta_j]e^{2\beta\lambda \sum_l \eta_l m_l^* [\eta_j - \eta_i]}$ – showing that fluctuations for low-centrality nodes are exponentially larger than for high-centrality nodes. Moreover, combining with Eq.(7-8) we find $\sigma(\mathbf{m}^*) \approx 1$ and

$$\frac{\mu_i(\mathbf{m}^*)}{\epsilon_i(\mathbf{m}^*)} = \frac{dp_i}{dm_i}(\mathbf{m}^*) \approx \frac{1}{1 - m_i^{*2}} = \frac{1}{4}e^{2\beta\lambda\eta_i \sum_l \eta_l m_l^*}. \tag{21}$$

For $\mathbf{m} \approx \mathbf{m}^*$, $S(\mathbf{m}) \approx \sum_i \int_{m_i^*}^{m_i} \frac{dp_i}{dm_i^{'}}(\mathbf{m}^*)(m_i^{'} - m_i^*)dm_i^{'}$, and thus

$$S(\mathbf{m}) \approx \sum_i -\frac{1}{8}(m_i - m_i^*)^2 e^{2\beta\lambda\eta_i \sum_l \eta_l m_l^*}, \tag{22}$$

which is equivalent to Eq.(14)(main text).

6 Finding the OP numerically

In general, one would like to find the OP beyond the limiting cases. Of course, no analytic solution is possible except in networks that are effectively low-dimensional. Since the path connects two equilibria via a heteroclinic orbit, in practice it must be constructed numerically, e.g., through shooting, or quasi-newton methods, etc. The method used in this report is of the latter form, namely the Iterative-Action-Minimizing-Method (IAMM) (doi:10.1016/j.phys d.2013.04.001). In this method, OPs are generated from a least-squares algorithm that minimizes the residuals between Eqs.(8-9)(main text) and finite-difference approximations, with fixed-point boundary conditions from Sec.(3.1)(main text) (used to close the differencing). However the dimension for the minimization is $2Nd$ where d is the number of discrete points in the differencing and N is the size of the network, which is prohibitively large for large N (typically we choose $200 \leq d \leq 500$). Therefore, in practice it is necessary to coarse-grain the network in some way. We describe our approach for this report in Sec.7. We mention that for the special case of $\mathbf{f} = \mathbf{0}$, the OP is *reversible*, and therefore $d\mathbf{m}/dt$ along the first segment is time reversed along the second.

7 Binning the network

We are interested in reducing the dimension of network defined by the adjacency, matrix, $A \in \mathcal{L}(\mathcal{R}^N, \mathcal{R}^N)$. All of the networks considered in this report are symmetric, though the formalism does not require this assumption. Nevertheless, in this section we assume $A = A^T$. Given a sequence of of eigenvalues, $\{\lambda_j\}$, and eigenvectors, $\{\boldsymbol{\eta}_j\}$, for A, we assume the largest eigenvalue is much greater than all of the others. This is a good approximation for many networks, including those discussed in Sec.3. From the spectral decomposition theorem, we can approximate the adjacency matrix as $A \approx \lambda \boldsymbol{\eta}\boldsymbol{\eta}^T$, where $\lambda = max\{\lambda_i\}$, and $\boldsymbol{\eta}$ the corresponding eigenvector.

In order to create a mapping from N dimensions to one that is significantly lower, we first notice that the entries of the eigenvector (which we assume is normalized) roughly relate a measure of vertex importance in the graph. Therefore, we reorder the entries of $\boldsymbol{\eta}$ in increasing order such that $\boldsymbol{v} = \boldsymbol{P}\boldsymbol{\eta}$, where $\boldsymbol{P} \in \mathcal{L}(\mathcal{R}^N, \mathcal{R}^N)$ is a permutation matrix, and $v_1 \leq v_2 \leq \cdots \leq v_N$. Notice we have not changed the norm of $\boldsymbol{v}$, nor have we made any dimension reduction.

Next, we arbitrarily pick a binning of $\boldsymbol{v}$ such that there exists $|B| \ll N$ bins, and associated with each bin $b \in B$ we have a distribution, g_b, as well as an index set, $\mathcal{I}_b$. We define an indicator function on an index such $\chi_b(i) = 1$ if $i \in \mathcal{I}_b$, 0 otherwise. We now define a vector that averages the nodes within a bin b as the following:

$$r_b = (1/Ng_b)[\chi_b(1), \chi_b(2), \ldots \chi_b(N)] \cdot \boldsymbol{v} \tag{23}$$

$$\equiv \boldsymbol{\alpha_b^T} \boldsymbol{v}. \tag{24}$$

That is, Ng_b is the total number of nodes in bin b, and $r_b = (1/Ng_b)\sum_{i \in \mathcal{I}_b} v_i$.

The map in Eq.23 computes the average over all of those nodes in bin b. To compute the entire transformation from $\mathcal{R}^N$ into $\mathcal{R}^B$,we have

$$\boldsymbol{r} = \begin{bmatrix} \boldsymbol{\alpha}_1^T & \cdot & \cdot & \cdot \\ \boldsymbol{\alpha}_2^T & \cdot & \cdot & \cdot \\ \vdots & \cdot & \cdot & \cdot \\ \boldsymbol{\alpha}_\beta^T & \cdot & \cdot & \cdot \end{bmatrix} \boldsymbol{P\eta} \equiv \boldsymbol{\mathcal{A}P\eta},$$

where $\boldsymbol{\mathcal{A}} \in \mathcal{L}(\mathcal{R}^N, \mathcal{R}^B)$. Using the same transformation map for $\mathbf{m}$ and $\mathbf{p}$, we find the corresponding $|B|$ dimensional vectors, $\boldsymbol{\mathcal{M}}$ and $\boldsymbol{\mathcal{P}}$, respectively, for the average opinion density and momentum in bins. By replacing v_i with r_b, m_i with $\mathcal{M}_b$, and p_i with $\mathcal{P}_b$ for $i \in \mathcal{I}_b$ in Eqs.(8-9)(main text), we get the (approximate) equations of motion for bin b:

$$\dot{\mathcal{M}}_b = \frac{(1-\mathcal{M}_b)e^{2\mathcal{P}_b}}{1+e^{-2\beta\lambda r_b \sum_{b'} N g_{b'} r_{b'} \mathcal{M}_{b'}}} - \frac{(1+\mathcal{M}_b)e^{-2\mathcal{P}_b}}{1+e^{2\beta\lambda r_b \sum_{b'} N g_{b'} r_{b'} \mathcal{M}_{b'}}} + f_b\left[(1-\mathcal{M}_b)e^{2\mathcal{P}_b} - (1+\mathcal{M}_b)e^{-2\mathcal{P}_b}\right], \quad (25)$$

$$\begin{aligned}\dot{\mathcal{P}}_b =& \frac{\frac{1}{2}(e^{2\mathcal{P}_b}-1)}{1+e^{-2\beta\lambda r_b \sum_{b'} N g_{b'} r_{b'} \mathcal{M}_{b'}}} - \frac{\frac{1}{2}(e^{-2\mathcal{P}_b}-1)}{1+e^{2\beta\lambda r_b \sum_{b'} N g_{b'} r_{b'} \mathcal{M}_{b'}}} + \frac{f_b}{2}\left[e^{2\mathcal{P}_b} - e^{-2\mathcal{P}_b}\right] \\ &- \beta\lambda r_b \sum_{b'} N g_{b'} r_{b'} \left[\frac{(1-\mathcal{M}_{b'})(e^{2\mathcal{P}_{b'}}-1) - (1+\mathcal{M}_{b'})(e^{-2\mathcal{P}_{b'}}-1)}{\left(e^{\beta\lambda r_{b'} \sum_{b''} N g_{b''} r_{b''} \mathcal{M}_{b''}} + e^{-\beta\lambda r_{b'} \sum_{b''} N g_{b''} r_{b''} \mathcal{M}_{b''}}\right)^2}\right], \end{aligned} \quad (26)$$

assuming $f_i = f_b \ \forall \ i \in \mathcal{I}_b$. A final requirement is needed to ensure that the binned and original system have the same bifurcation point and are similarly normalized: after binning we *renormalize* r_b so that $\sum_j \eta_j^2 = \sum_b r_b^2 g_b N = 1$.

In practice, to use the binning procedure one must specify $\chi_b(i)$. We illustrate with the Facebook network, where we chose $|B| = 50$. The v_i distribution is shown in Fig.1 in blue. Note, the first 3000 nodes have small v_i, and therefore we truncate the x-axis for easier viewing. Visually the v_i has roughly three relevant parts: $v_i \sim \mathcal{O}(0.1)$, $\mathcal{O}(0.01)$, and $\mathcal{O}(0.001)$ or smaller. The binned distribution is shown in red. We chose to bin

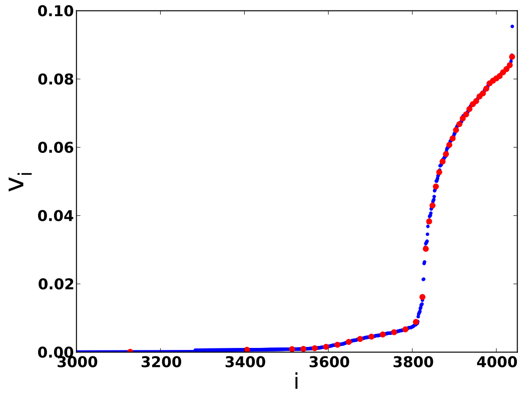

Figure 1: Example binning procedure for Facebook network. Eigenvector centralities (blue) are shown for all network positions and compared with the average centrality in each bin (red)

each of the three parts with roughly equal numbers of nodes in each bin – with a total of 28, 12, and 10 bins for the three parts, respectively. Such a choice gave the following indicator functions, $\chi_b(i)$, which we list in

their entirety for completeness:

$$\begin{aligned}
\chi_1(i) &= 1 \text{ if } 0.09541 < v_i, & \chi_{26}(i) &= 1 \text{ if } 0.03454 < v_i \leq 0.04072,\\
\chi_2(i) &= 1 \text{ if } 0.08605 < v_i \leq 0.09541, & \chi_{27}(i) &= 1 \text{ if } 0.02598 < v_i \leq 0.03454,\\
\chi_3(i) &= 1 \text{ if } 0.08352 < v_i \leq 0.08605, & \chi_{28}(i) &= 1 \text{ if } 0.01309 < v_i \leq 0.02598,\\
\chi_4(i) &= 1 \text{ if } 0.08226 < v_i \leq 0.08352, & \chi_{29}(i) &= 1 \text{ if } 0.00722 < v_i \leq 0.01309,\\
\chi_5(i) &= 1 \text{ if } 0.08155 < v_i \leq 0.08226, & \chi_{30}(i) &= 1 \text{ if } 0.00624 < v_i \leq 0.00722,\\
\chi_6(i) &= 1 \text{ if } 0.08040 < v_i \leq 0.08155, & \chi_{31}(i) &= 1 \text{ if } 0.00556 < v_i \leq 0.00624,\\
\chi_7(i) &= 1 \text{ if } 0.08001 < v_i \leq 0.08040, & \chi_{32}(i) &= 1 \text{ if } 0.00488 < v_i \leq 0.00556,\\
\chi_8(i) &= 1 \text{ if } 0.07913 < v_i \leq 0.08001, & \chi_{33}(i) &= 1 \text{ if } 0.00430 < v_i \leq 0.00488,\\
\chi_9(i) &= 1 \text{ if } 0.07800 < v_i \leq 0.07913, & \chi_{34}(i) &= 1 \text{ if } 0.00347 < v_i \leq 0.00430,\\
\chi_{10}(i) &= 1 \text{ if } 0.07682 < v_i \leq 0.07800, & \chi_{35}(i) &= 1 \text{ if } 0.00242 < v_i \leq 0.00347,\\
\chi_{11}(i) &= 1 \text{ if } 0.07555 < v_i \leq 0.07682, & \chi_{36}(i) &= 1 \text{ if } 0.00191 < v_i \leq 0.00242,\\
\chi_{12}(i) &= 1 \text{ if } 0.07433 < v_i \leq 0.07555, & \chi_{37}(i) &= 1 \text{ if } 0.00130 < v_i \leq 0.00191,\\
\chi_{13}(i) &= 1 \text{ if } 0.07287 < v_i \leq 0.07433, & \chi_{38}(i) &= 1 \text{ if } 0.00103 < v_i \leq 0.00130,\\
\chi_{14}(i) &= 1 \text{ if } 0.07234 < v_i \leq 0.07287, & \chi_{39}(i) &= 1 \text{ if } 0.00091 < v_i \leq 0.00103,\\
\chi_{15}(i) &= 1 \text{ if } 0.07038 < v_i \leq 0.07234, & \chi_{40}(i) &= 1 \text{ if } 0.00086 < v_i \leq 0.00091,\\
\chi_{16}(i) &= 1 \text{ if } 0.06939 < v_i \leq 0.07038, & \chi_{41}(i) &= 1 \text{ if } 0.000610 < v_i \leq 0.00086,\\
\chi_{17}(i) &= 1 \text{ if } 0.06743 < v_i \leq 0.06939, & \chi_{42}(i) &= 1 \text{ if } 1.452329E{-}5 < v_i \leq 0.000610,\\
\chi_{18}(i) &= 1 \text{ if } 0.06652 < v_i \leq 0.06743, & \chi_{43}(i) &= 1 \text{ if } 3.145425E{-}6 < v_i \leq 1.452329E{-}5,\\
\chi_{19}(i) &= 1 \text{ if } 0.06404 < v_i \leq 0.06652, & \chi_{44}(i) &= 1 \text{ if } 1.9930885E{-}6 < v_i \leq 3.145425E{-}6,\\
\chi_{20}(i) &= 1 \text{ if } 0.06209 < v_i \leq 0.06404, & \chi_{45}(i) &= 1 \text{ if } 2.4888568E{-}7 < v_i \leq 1.9930885E{-}6,\\
\chi_{21}(i) &= 1 \text{ if } 0.05991 < v_i \leq 0.06209, & \chi_{46}(i) &= 1 \text{ if } 9.2757062E{-}08 < v_i \leq 2.4888568E{-}7,\\
\chi_{21}(i) &= 1 \text{ if } 0.05695 < v_i \leq 0.05991, & \chi_{47}(i) &= 1 \text{ if } 5.9389469E{-}08 < v_i \leq 9.2757062E{-}08,\\
\chi_{22}(i) &= 1 \text{ if } 0.05484 < v_i \leq 0.05695, & \chi_{48}(i) &= 1 \text{ if } 2.2296092E{-}08 < v_i \leq 5.9389469E{-}08,\\
\chi_{23}(i) &= 1 \text{ if } 0.05095 < v_i \leq 0.05484, & \chi_{49}(i) &= 1 \text{ if } 6.5813248E{-}10 < v_i \leq 2.2296092E{-}08,\\
\chi_{24}(i) &= 1 \text{ if } 0.04556 < v_i \leq 0.05095, & \chi_{50}(i) &= 1 \text{ if } 6.2455171E{-}14 < v_i \leq 6.5813248E{-}08,\\
\chi_{25}(i) &= 1 \text{ if } 0.04072 < v_i \leq 0.04556, & &\text{and } \chi_b(i) = 0 \text{ otherwise } \forall b.
\end{aligned}$$

Note, $g_b = \sum_i \chi_b(i)/N$. Similar binning procedures were used for all other networks, Sec.3.

8 Control

For the first control (Sec.3.3, 2nd paragraph, main text), the control set F with the largest $\langle\eta\rangle_F$ corresponds to the first 4 bins (centered on red points) starting from the right in Fig.1. In particular, the control set contains 32 nodes with the highest v_i, or the first 32 blue points starting from the right in Fig.1. The next control set with the second largest $\langle\eta\rangle_F$ corresponds to bins $5-8$ starting from the right in Fig.1 – namely, the next 32 nodes with highest centrality but less than the lowest centrality in the first set of 32 nodes. This pattern is continued for six different control sets and three flipping rates, f, Fig.4(b)(main text).

For the second control (Sec.3.3, 3rd paragraph, main text), again we start with the 32 nodes with highest v_i as our control set, and then add/subtract nodes with lower/higher v_i. For example, moving one point to the left along the $|F|$ axis in Fig.4(c)(main text) to $|F|=24$, implies controlling 24 nodes with the highest v_i. Moving, one point to the right implies controlling 40 nodes with the highest v_i, and so forth. Since the size of the control set is changed, we change f in order to keep a quantity constant. The two constants chosen for Fig.4(c)(main text) were $f|F|$ (blue points) and $\sum_i m_i^{*2}$ (green diamonds). We found little change in the Action when the order was held constant. We mention that this is not always the case: in epidemics, minimizing the epidemic size does not imply minimizing the Action in general (see ref.[15], main text).
